# Supplementary material for: Antimicrobial Activity of Sertraline on Listeria monocytogenes
Source: Int J Mol Sci. 2023 Feb 28;24(5):4678. doi: 10.3390/ijms24054678 (PMC10002541; doi:10.3390/ijms24054678)
Supplement: Supplementary file 1 [file ijms-24-04678-s001.zip › ijms-2166742-SI.pdf]

Supplementary Material

Supplementary Figure S1. The relationship between percentage of living cells and relative fluorescence intensity. (A) *Listeria monocytogenes* ATCC 11915; (B) *Listeria monocytogenes* 001.

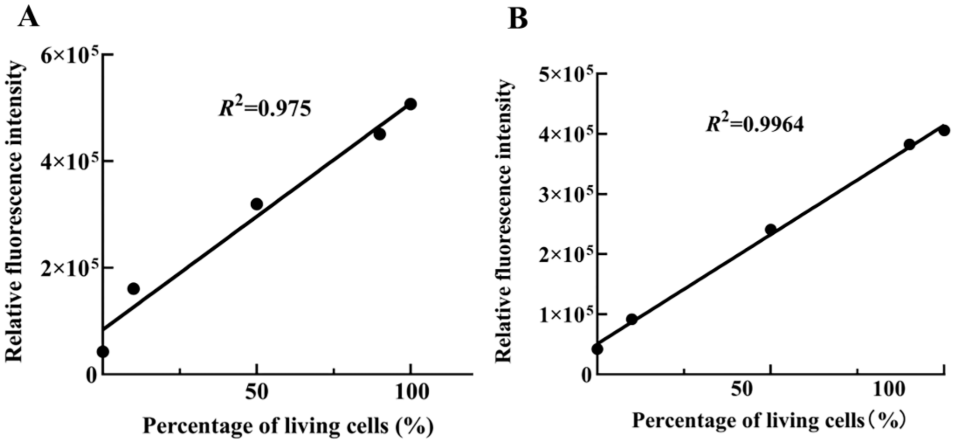

Table S1. Primers used in quantitative real-time PCR transcriptional analysis.

Gene	Nucleotide sequence	Amplicon size (bp)
hly	5′AGCTCATTTCACATCGTCCA 3′	124
	5′TGGTAAGTTCCGGTCATCAA 3´	
agrA	5′CGGGTACTTGCCTGTATGAA 3′	149
	5′TGAATAGTTGGCGCTGTCTC 3′	
degU	5′GGCGCGTATATTCATCCAC 3′	150
	5′TACCTCGCACTCTCTATGCG 3′	
prfA	5′GGAAGCTTGGCTCTATTTGC 3′	145
	5′ACAGCTGAGCTATGTGCGAT 3′	
actA	5′AGAAATCATCCGGGAAACAG 3′	147
	5′CCTCTCCCGTTCAACTCTTC 3′	
flaA	5′GTAAGCATCCAAGCGTCTGA 3′	148
	5′AAGAATCAGCATCAGCAACG 3′	
sigB	5′TGGTGTCACGGAAGAAGAAG 3′	135
	5′TCCGTACCACCAACAACATC 3′	
ltrC	5′TACGGCGTCGAT ATACT 3′	144
	5′GAATGTGTGAACGGCGATAC 3′	
sufS	5′GAATTTGGCGGAGAAATGAT 3′	137
	5′TCTGCCAAGTAATCAATCGC 3′	
sufU	5′TTCAGAAATGGTGCAAGGTC 3′	135
	5′ATCGCTCTCTCCATTGCTTT 3′	
16S RNA	5′TTAGCTAGTTGGTAGGGT 3′	550
	5′AATCCGGACAACGCTTGC 3′	

-
